# A 38-year-old woman with zosteriform skin lesions

**DOI:** 10.1371/journal.pntd.0005906

**Published:** 2017-11-02

**Authors:** Daniel Camprubí, Ana Requena-Méndez, Natalia Rodríguez, M. Eugenia Valls, Adriana García-Herrera, Teresa Estrach, Xavier Fustà, Juan García-Bernalt Diego, Pedro Fernández-Soto, Jose Muñoz

**Affiliations:** 1 ISGlobal, Barcelona Ctr. Int. Health Res. (CRESIB), Hospital Clínic-Universitat de Barcelona, Barcelona, Spain; 2 Microbiology and Parasitology, Hospital Clínic, Barcelona, Spain; 3 Pathology Department, Hospital Clinic, Barcelona, Spain; 4 Dermatology Department, Hospital Clinic, Barcelona, Spain; 5 Infectious and Tropical Diseases Research Group (e-INTRO), Biomedical Research Institute of Salamanca-Research Centre for Tropical Diseases at the University of Salamanca (IBSAL-CIETUS), University of Salamanca, Salamanca, Spain; George Washington University School of Medicine and Health Sciences, UNITED STATES

## Question

**Fig 1:**
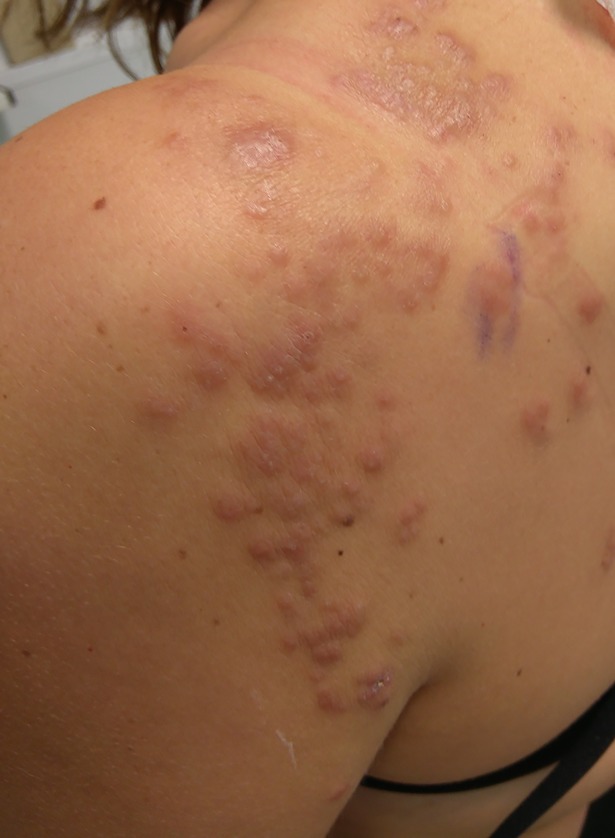
Clinical image. Pruritic painless erythematous papules grouped on the back part of the left shoulder of the patient following the C3-C4 dermatome.

**Fig 2:**
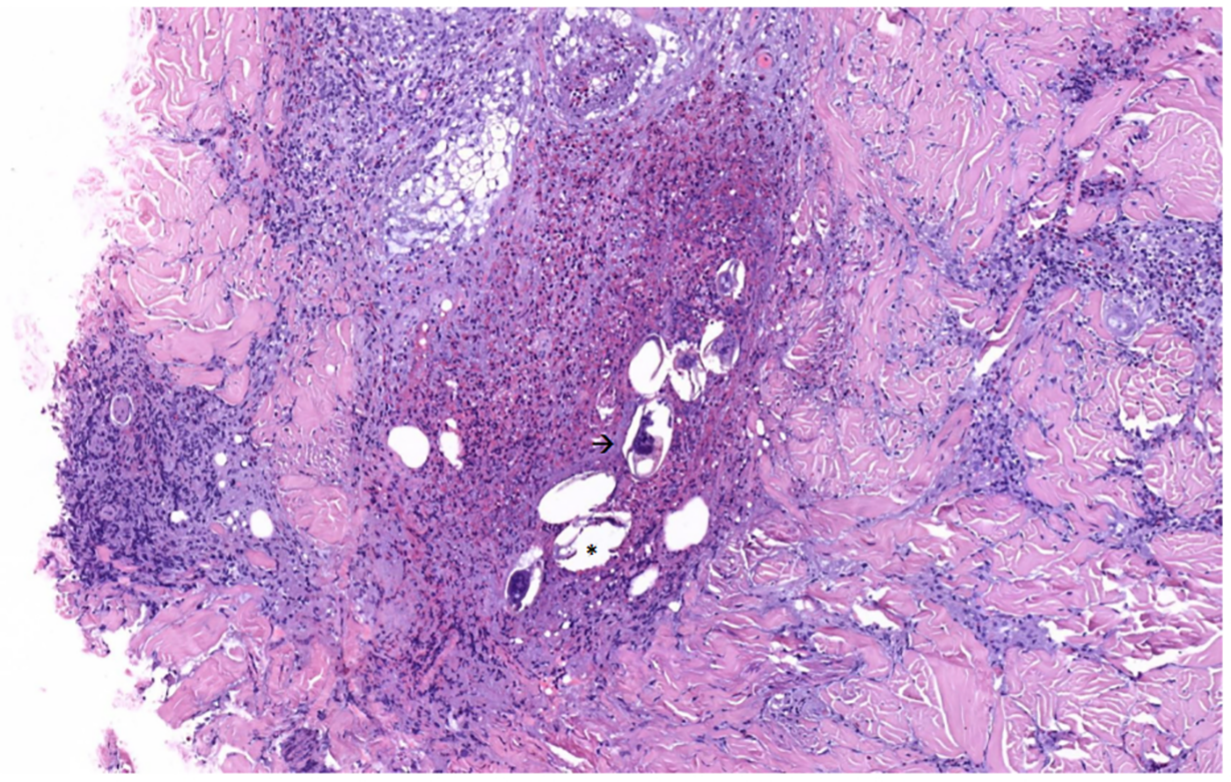
Pathological image. Dermal inflammatory infiltrates composed of lymphocytes, eosinophils, and histiocytes involving multiple eggs with lateral spine (asterisk) and miracidia inside (arrow).

A 38-year-old white woman with no previous medical history presented to our hospital with a 7-day clinical history of skin lesions on her shoulder. She had visited Uganda for 3 weeks, where she swam in Bunyonyi Lake at the end of the first week of the trip. She remained asymptomatic until 2 weeks after her return (approximately 5 weeks after freshwater exposure), when she developed pruritic painless erythematous papules grouped on the back part of her left shoulder following the C3-C4 dermatome (Fig 1). Under clinical suspicion of cutaneous herpes zoster, oral valganciclovir was prescribed. No clinical improvement was observed, and varicella zoster virus polymerase chain reaction from a skin lesion was negative. Routine blood tests showed eosinophilia (5,200 cells/mm^3^, normal count below 450 cells/mm^3^). No parasites, cysts, or eggs were observed in excreta. Two weeks after the clinical symptoms appeared and while waiting for the results of some serological tests, a skin biopsy (Fig 2) and a specific loop-mediated isothermal amplification (LAMP) assay of the sample were performed. What is your diagnosis?

## Diagnosis

### Early ectopic cutaneous schistosomiasis

Pathological examination of the skin showed dermal inflammatory infiltrates composed of lymphocytes, eosinophils, and histiocytes including multiple eggs with lateral spine and miracidia inside (Fig 2), and Ziehl-Neelsen staining was positive [1]. An indirect hemagglutination test (Schistosomiasis Fumouze) detected high titers (1/640, titers above 1/80 are considered positive) of *Schistosoma* antibodies. A diagnosis of ectopic cutaneous schistosomiasis was made, and treatment with prednisone 0.5 mg/kg/day and a single dose of praziquantel (40 mg/kg) was started. In the next few days, the patient’s eosinophil count decreased progressively, and cutaneous lesions partially improved. Given the particular chronology of the case, the patient was asked about previous travel to endemic areas of schistosomiasis and previous freshwater exposure during travel, but there were none. Finally, the suspicion of *S*. *mansoni* as the most likely etiological agent, considering the epidemiological history and the histological findings on the skin biopsy, was confirmed by a species-specific LAMP-based real-time assay.

A molecular LAMP assay previously developed for sensitive and specific *S*. *mansoni* detection (SmMIT-LAMP) [2] was performed using the patient’s skin tissue sample. Briefly, DNA from a formalin-fixed paraffin-embedded biopsy was isolated using the QIAamp DNA Mini Kit (QIAgen, Hilden, Germany) following manufacturer instructions. The isothermal SmMIT-LAMP master mixtures—including the patient’s sample and controls—were, in this case, monitored using 2 fluorochromes to compare (SYBR Green I and EvaGreen) in real time at 65°C for 60 minutes using the portable Genie III instrument (Optigene Ltd., Horsham, UK). Amplification results were observed in positive control samples (genomic DNA extracted from *S*. *mansoni*) as well as in the patient's tissue sample when using EvaGreen dye (0.5x). There was 100% concordance with the results on agarose gel electrophoresis.

## Discussion

Human schistosomiasis is a parasitic disease caused by several species of *Schistosoma*, affecting more than 200 million people worldwide [3]. After skin penetration of cercariae, adult worms infect human blood vessels for years, predominantly from mesenteric and perivesical plexus, depending on the *Schistosoma* species. They can successfully evade the immune system while excreting fertilized eggs that will leave the body in feces or urine to reach freshwater and infect snails to complete their cycle, or they can become trapped in nearby tissues [3].

Two different clinical pictures of *Schistosoma* spp. infection have been described, depending on the immunity of their hosts. Acute schistosomiasis—also called Katayama syndrome—is considered an immune reaction to the *Schistosoma* larvae and/or eggs that affects nonimmune individuals and requires treatment with corticosteroids alone or in addition to praziquantel [4]. On the other hand, chronic schistosomiasis is the result of an immune-mediated granulomatous response to trapped eggs in immune local patients that produces organ-specific manifestations, such as hematuria, obstructive uropathology, hepatosplenism, periportal fibrosis with portal hypertension, and neurological symptoms [3].

Skin lesions caused by this trematode can be classified depending on their chronopathology as immediate, early, and chronic. The first one, also known as swimmer’s itch or cercarial dermatitis, is an immediate skin manifestation characterized by a generalized pruritic papular rash that appears as an immune reaction when cercariae penetrate the skin. Early skin lesions are allergic reactions (usually urticaria) produced in the context of Katayama syndrome [4]. Finally, classical chronic cutaneous schistosomiasis consists of papular, erythematous, and pruritic lesions located in the anogenital region, and only a few cases of extragenital skin lesions have been described [5–9]. Therefore, ectopic cutaneous schistosomiasis is characterized by erythematous pruritic papules with a zosteriform distribution that can evolve to nodules and plaques. It mainly affects the thorax—particularly around the shoulders—and abdomen, and it has been reported more frequently in nonimmune travelers visiting endemic areas [7].

The differential diagnosis of chronic cutaneous schistosomiasis includes sexually transmitted diseases and neoplasms due to its usual perigenital distribution. In cases of ectopic schistosomiasis alternative diagnoses like subcutaneous tuberculosis and tuberculoid leprosy, more frequently diagnosed in people from endemic areas, must be considered. Furthermore, in our case (given the distribution of the lesions), the differential diagnosis must include viral infections like herpes zoster or zosteriform infections by herpes simplex virus, bacterial infections like erisipela or anthrax, contact dermatitis, phytodermatosis, and sarcoidosis.

As in our case, eosinophilia is a usual laboratory finding, and histopathological examination of the lesions shows granulomas around *Schistosoma* eggs and inflammatory infiltrates of eosinophils and lymphocytes. The mechanism of ectopic oviposition is unknown, but some theories have been proposed. Some authors suggested the unlikely existence of arteriovenous shunts like foramen ovale, but probably the most accepted hypothesis is the aberrant migration of adult worms against the venous blood stream. *Schistosoma* worms ascend from iliac and cava veins through lumbar veins and valveless vertebral venous circulation to paraspinal and intercostals cutaneous locations. That could entail the deposition of eggs in the skin following a zosteriform distribution [8]. Although ectopic skin lesions usually occur independently, an association with some severe clinical coexisting manifestations such as myelitis has been reported in a few cases [9]. Therefore, treatment is important not only to eliminate the adult worms and halt the oviposition but also to avoid the evolution to severe forms [9].

A diagnosis of acute schistosomiasis is usually based on a combination of clinical and epidemiological factors. In our case, we used, for the first time, a specific real-time LAMP assay based on a skin biopsy to confirm the diagnosis and to determine the *S*. *mansoni* species causing the cutaneous lesions as a proof of concept of the usefulness of LAMP in the diagnosis of acute schistosomiasis.

Finally, although ectopic cutaneous schistosomiasis has been classified as a manifestation of chronic and/or late schistosomiasis—usually described as occurring 3 months after water exposure—physicians must be aware of early presentations, as in the reported case of our patient, who started having cutaneous manifestations only 5 weeks after her exposure to the infected freshwater. Despite differences in treatment, there is no clear consensus about definitions of acute and chronic schistosomiasis. At this point, we consider that new definitions of chronic and acute schistosomiasis should be established, based not only on the chronology but also on the physiopathological aspects. Furthermore, it is important to highlight the role of LAMP assays in the identification of *Schistosoma* species and their potential importance in the diagnosis of schistosomiasis.

### Statement of agreement

The patient agreed to the publication of pictures and information related with the case. She signed an informed consent form.

**Key learning points**

- Cutaneous zosteriform lesions in a person with the appropriate epidemiological risk factors can be a clue for the diagnosis of schistosomiasis.
- Ectopic cutaneous lesions can appear in early stages of the disease.
- New molecular techniques such as LAMP can help in the diagnosis of schistosomiasis.
- Treatment of cutaneous schistosomiasis is important to avoid progression to severe forms.
- New definitions of chronic and acute schistosomiasis should be considered on the basis of the chronology and physiopathological aspects.
